# Accelerated epigenetic aging in Down syndrome

**DOI:** 10.1111/acel.12325

**Published:** 2015-02-09

**Authors:** Steve Horvath, Paolo Garagnani, Maria Giulia Bacalini, Chiara Pirazzini, Stefano Salvioli, Davide Gentilini, Anna Maria Di Blasio, Cristina Giuliani, Spencer Tung, Harry V Vinters, Claudio Franceschi

**Affiliations:** 1 Human Genetics, David Geffen School of Medicine, University of California Los AngelesLos Angeles, CA, 90095, USA; 2 Biostatistics, Fielding School of Public Health, University of California Los AngelesLos Angeles, CA, 90095, USA; 3 Department of Experimental, Diagnostic and Specialty Medicine, University of BolognaBologna, 40126, Italy; 4 Interdepartmental Center ‘L. Galvani’, University of BolognaBologna, 40126, Italy; 5 CNR, Applied Biomedical Research Center, S. Orsola-Malpighi PolyclinicBologna, 40138, Italy; 6 Personal Genomics S.r.l.Verona, 37134, Italy; 7 Center of Research and Biomedical Technology, Istituto Auxologico Italiano IRCCSVia Zucchi 18, Cusano Milanino, 20095, Milan, Italy; 8 Department of Biological, Geological and Environmental Sciences, University of BolognaBologna, 40126, Italy; 9 Department of Neurology and Department of Pathology and Laboratory Medicine, David Geffen School of Medicine at UCLALos Angeles, CA, 90095, USA; 10 IRCCS, Institute of Neurological Sciences of Bologna40139, Bologna, Italy

**Keywords:** biomarker of aging, DNA methylation, Down syndrome, epigenetics

## Abstract

Down Syndrome (DS) entails an increased risk of many chronic diseases that are typically associated with older age. The clinical manifestations of accelerated aging suggest that trisomy 21 increases the biological age of tissues, but molecular evidence for this hypothesis has been sparse. Here, we utilize a quantitative molecular marker of aging (known as the epigenetic clock) to demonstrate that trisomy 21 significantly increases the age of blood and brain tissue (on average by 6.6 years, *P *= 7.0 × 10^−14^).

## Introduction

Down Syndrome (DS) is a genetic disorder caused by the presence of all or part of a third copy of chromosome 21 (trisomy 21; +21). It is not only associated with intellectual disability but also with a group of clinical manifestations of ‘accelerated aging’ (Devenny *et al*., 2005; Patterson & Cabelof, 2012; Moran, 2013). Adults with DS have a specific list of conditions with accelerated age at onset, including premature skin wrinkling, greying of hair, hypogonadism, early menopause, hypothyroidism, declining immune function, and Alzheimer's disease. However, as recently reviewed (Zigman, 2013), accelerated aging in DS is atypical and segmental, involving some but not all organs and tissues. Of particular interest are the brain (Lott & Head, 2005; Teipel & Hampel, 2006) and the immune system (Cuadrado & Barrena, 1996). Certain functional abnormalities of lymphocytes can be interpreted as precocious immunosenescence (Kusters *et al*., 2011) but alternative interpretations are possible (Cuadrado & Barrena, 1996).

Due to the limited availability of biomarkers of aging, it has been difficult to rigorously test whether DS is associated with accelerated aging effects, per se, versus specific vulnerabilities to conditions typically associated with old age (e.g., increased risk for Alzheimer's disease). One basic tenet is that a suitable biomarker of aging should detect accelerated aging effects in tissues (e.g., brain and blood) that exhibit clinical manifestations of accelerated aging in DS subjects.

Telomere length can be used as a molecular aging marker and (Vaziri *et al*., 1993) reported that DS cases exhibited shorter leukocyte telomere length (LTL) than control subjects, with more recent data suggesting that shorter telomere length is associated with the presence of both dementia and mild cognitive impairment in adults with DS (Jenkins *et al*., 2008). However, one study found no evidence that cultured skin fibroblasts from DS subjects attained replicative senescence (a telomere length-dependent phenomenon) earlier than those from controls (Kimura *et al*., 2005).

Here, we study a new type of molecular marker of aging, our recently developed ‘epigenetic clock’, which is based on DNA methylation (DNAm) levels (Horvath, 2013). The epigenetic clock has several advantages over other methods. It is quantitative, it overcomes the technical difficulties that plague telomere length measurements and is applicable in all types and sources of tissues, importantly including cryopreserved banked specimens (Horvath, 2013). This epigenetic clock, which we defined by studying DNAm in series of human tissues across a range of chronological ages, is a prediction method of chronological age based on the DNAm levels of 353 specific CpGs. In normal individuals, the predicted (estimated) age is referred to as DNAm age. DNAm age is highly correlated with chronological age across sorted cell types (CD4 T cells, monocytes, neurons), complex tissues (e.g., blood, brain, liver) (Horvath, 2013; Horvath *et al*., 2014). The epigenetic clock can be applied to two commercially standardized methylation platforms: the Illumina 450K and the 27K arrays.

Our 4 DNAm data sets are described in Table1 and in the Methods. DNA methylation levels were assessed in peripheral blood leukocytes (data set 1; 27K array), various brain regions (from DS subjects, Alzheimer's disease subjects and controls, data set 2; 450K array), whole blood (data set 3; 450K array) and buccal epithelium (data set 4; 27K arrays).

**Table 1:** Overview of the DNA methylation data sets

Tissue source	Platform	N (total)	Perc. female, %	N (DS)	Mean Age (range)	GEO ID	Citation	*P*-value
1. Leukocytes	27K	56	70	35	43 (22, 64)	GSE25395	Kerkel *et al*. (2010)	0.0048
2. Brain	450K	71	47	15	53 (32, 64)	GSE63347	Current article	5.5E-7
3. Whole blood	27K	87	63	29	33 (9,83)	GSE52588	Current article	0.00056
4. Buccal epithelium	27K	20	50	10	34 (27, 47)	GSE50586	Jones *et al*. (2013b)	0.26

The rows correspond to the data sets used in this article. Columns report the tissue source, DNAm platform, total number of samples (N), percent female, number of DS samples, mean age (and range, i.e., minimum age, maximum age), data access information, and citation. The last column reports a (Kruskal–Wallis test) *P*-value regarding the relationship between age acceleration and DS status. Additional details can be found in the Supporting Information.

**Fig 1:**
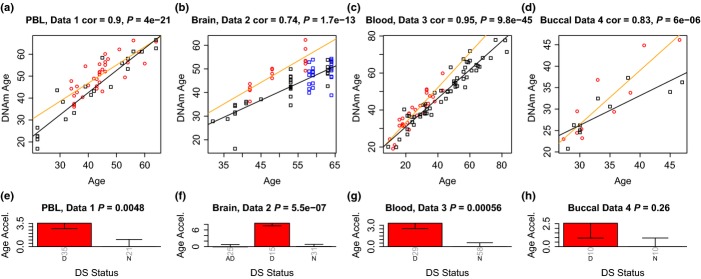
Analysis of 4 independent Down syndrome data sets. Each column corresponds to a different data set. The top row (a–d) shows scatter plots between chronological age (x-axis) and DNAm age (y-axis) in the 4 independent data sets. Red circles indicate DS, blue circles indicate Alzheimer's disease, and black squares denote control subjects. The orange line corresponds to a regression line through DS subjects. The black line in the upper panels indicates the regression line through the remaining (control) samples. The age acceleration effect for each subject (point) corresponds to the vertical distance to the black regression line. While DNAm age is highly correlated with chronological age, red points tend to lie above the black line, which indicates that DS subjects exhibit accelerated aging effects. The bottom row (e–h) show how mean age acceleration (y-axis) relates to DS status. By definition, the mean age acceleration measure in controls is zero. The title of the bar plots also reports a *P*-value from a nonparametric group comparison test (Kruskal Wallis test). Each bar plot reports 1 SE.

As expected, DNAm age has a strong positive correlation with chronological age in the control samples (0.74 < r < 0.95, Fig.1a–d). Therefore, we were able to define a measure of age acceleration as the residual resulting from a linear model that regressed DNAm age on chronological age in controls. By construction, this measure of age acceleration is not correlated with chronological age and takes on a positive value for samples whose DNAm age is higher than expected.

Strikingly, we find that DS subjects exhibit a highly significant age acceleration effect in three independent data sets (*P* = 0.0048, *P* = 5.5×10^-7^, *P* = 0.00056 Fig.1e–g) involving blood and brain tissue. By contrast, we did not observe a significant age acceleration effect in buccal epithelium (*P* = 0.26, Fig.1h) which might reflect that clinical manifestations of accelerated aging have not been observed in this tissue. However, we caution the reader that data set 4 only involved 10 cases and 10 controls. Larger studies may succeed at detecting an accelerated aging effect in buccal epithelium.

A meta-analysis applied to the blood and brain data sets shows that DS status is highly significantly (*P* = 7.0 × 10^−14^) associated with increased DNAm age (average 6.6 years, Table2).

**Table 2:** Estimation of the influence of DS on DNAm age

Variable	Data set 1		Data set 2		Data set 3		Data set 4	
	Leukocytes		Brain		Whole blood		Buccal Epithel.	
	Estimate (SE)	*P*	Estimate (SE)	*P*	Estimate (SE)	*P*	Estimate (SE)	*P*
Chronological age	0.95374 (0.06191)	<2 × 10^−16^	0.74144 (0.0565)	<2 × 10^−16^	0.79168 (0.02865)	<2 × 10^−16^	0.9452 (0.1449)	5.2 × 10^−6^
DS	3.74593 (1.37252)	8.6 × 10^−3^	8.53045 (1.23934)	2.3 × 10^−9^	3.65564 (1.08842)	1.2 × 10^−3^	2.6495 (1.7776)	0.15
R-squared	0.83		0.73		0.92		0.72	
Age accel. for DS	3.9 years		11.5 years		4.6 years		2.8 years	

Using a multivariate regression model DNAm age is regressed on chronological age and DS status. The table reports estimates of the regression coefficients and corresponding standard errors, Wald test *P*-values. The last row reports the age acceleration associated with DS status. For example DS status is associated with an increase of 3.9 years (=3.74593/0.95374) in the first data set. The results for data sets 1–3 are largely unchanged after including blood cell type abundance measures as additional covariates (Table s1).

Data set 2 shows that the brain tissue of DS subjects exhibits significantly higher age acceleration than that of AD subjects or controls (Fig.1f). These results can also be found when restricting attention to samples from the cerebellum, frontal lobe, or other brain regions (Fig. s1).

Data set 3 involved blood from 29 individuals with DS, their mothers and their unaffected siblings. This design allows adjustment for possible confounding effects on DNA methylation patterns deriving from genetic and environmental (lifestyle) factors within families. To properly account for the family relationships, we also fit a mixed effects model to the 29 discordant sib pairs. Specifically, DNAm age was regressed on DS status, chronological age, and a random effects term (intercept) that encoded the sib ship. Using this model, we found that DS status significantly affected DNAm age (*P* = 0.00029), raising it by 3.86 years ± 1.03 (SE).

Additional analyses examined potential confounders of gender and mean methylation levels. None of these factors contributed significantly to the effect of DS on DNAm age (e.g., Figs S2 and S3, Data S1).

Several studies reported age-dependent defects in the innate and in the adaptive immune system of DS (Kusters *et al*., 2011), consisting in altered prevalence of the different lymphocyte subpopulations. In data sets 1, DS status is not significantly associated with blood cell type proportions (Fig. s4m–r). But using the discordant sib pair data from data set 3, we observe that DS subjects exhibit fewer CD4 T cells and B cells (Fig. s5n,p) and more Natural Killer cells (Fig. s5o). Considering that DNA methylation is cell type specific, differences in blood cell counts could confound the relationship between DS status and age acceleration effects. While no significant correlation can be found between proportions of blood cell types and age acceleration in data set 1 (Fig. s4a–f), we observe a significant negative correlation for CD4 T cells (Fig. s5b) and monocytes (Fig. s5e). But differences in blood cell counts do not explain the observed age acceleration effects as can be seen from the fact that DS status remains a significant predictor of DNAm age in a multivariate regression model that includes blood cell counts and chronological age (Table s1). Similarly, the results remain significant (Table s2) when using a ‘reference free’ method that does not require reference libraries of cell types (Houseman *et al*., 2014). Further, a structural equation model analysis shows that causal models that posit that changes in blood cell type abundance mediate the effect of DS on age acceleration do not fit our data (Table s3).

Our observed results regarding the relationship between DS status and epigenetic age acceleration do not depend on any particular choice of aging signature (Fig. s6). In particular, they can also be found using an alternative age predictor described in (Hannum *et al*., 2013), and using aging signatures from (Teschendorff *et al*., 2010) and (Horvath *et al*., 2012).

A marginal analysis that related individual CpGs to age, acceleration, and DS status can be found in Figs S7–10 and 2. As the epigenetic clock was constructed as a multivariate predictor, it is not surprising that most of the 353 clock CpGs do not exhibit a consistent age correlation across data sets (Fig. s7a–c). These results demonstrate that the total (epigenetic clock) is more than the sum of its parts. The marginal association of CpGs with epigenetic age acceleration is only moderately (*r* = 0.41) preserved between data sets 1 and 2 (Fig. s7d–f). Similarly, marginal associations with DS status are moderately preserved across the blood and brain data sets (Fig. s8a–c, e–f).

**Fig 2:**
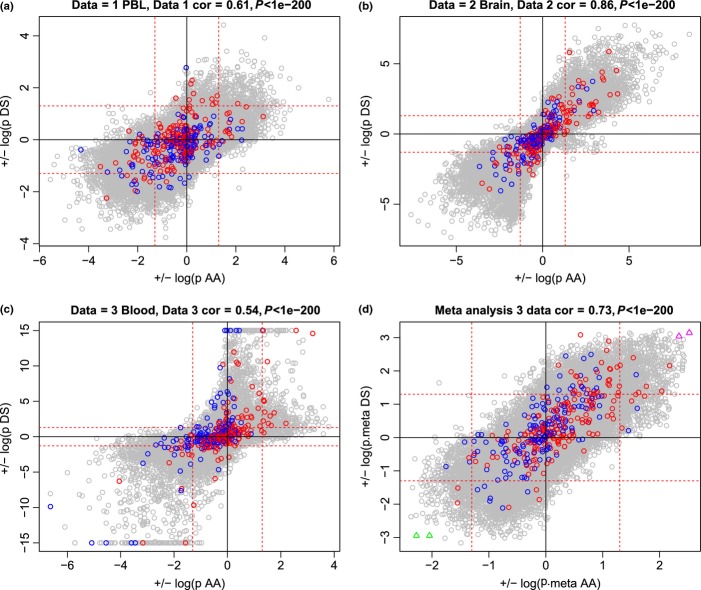
CpGs that relate to DS status tend to be correlated with age acceleration. (a–c) Results for data sets 1–3, respectively. d) Results of a meta-analysis (Supplementary Information). Each x-axis reports the marginal association with epigenetic age acceleration (as detailed in Supplementary Information and Fig. s7). The sign of the log (base 10) transformed *P*-value was chosen so that a positive (negative) value indicates that the CpG has a positive (negative) correlation with age acceleration. Each y-axis reports the marginal association with DS status (as detailed in Fig. s8). The sign of the log-transformed *P*-value was defined such that a positive value indicates that the CpG is hypermethylated in DS subjects. The 353 clock CpGs are colored according to their age correlation in the original training data set from (Horvath, 2013): red and blue for clock CpGs that have a positive and negative age correlation, respectively. The red horizontal and vertical lines correspond to an uncorrected *P*-value threshold of 0.05.

Strikingly, CpGs that are associated with DS status tend to be correlated with epigenetic age acceleration (Fig.2). For example, we present CpGs that are hypermethylated in DS subjects and have a positive correlation with age acceleration in Fig. s9. These two CpGs correspond to the two magenta triangles in the upper right hand corner of Fig.2d. It is noteworthy that CpG cg12186917 is located on chromosome 21 (near gene CRYZL1). The other CpG cg04455759 is close to gene MRPL42 (mitochondrial ribosomal protein L42) on chromosome 12. We also present two CpGs that are both hypomethylated in DS subjects and have a negative correlation with age acceleration (Fig. s10). These CpGs correspond to the green triangles in the lower left corner of Fig.2d. CpG cg11678767 is located near COL4A6 (type IV alpha 6 collagen isoform A precursor) on the X chromosome, whereas CpG cg20425293 is located near gene SDHD (succinate dehydrogenase complex; subunit D precursor) on chromosome 11. Results for individual CpGs can be found in the Supporting File. A detailed marginal analysis is beyond our scope but we briefly point out that it is already known that DS does not affect global methylation levels in blood (Kerkel *et al*., 2010) or buccal epithelium (Jones *et al*., 2013a).

Due to a lack of transcriptional data, we could not directly correlate epigenetic age acceleration with gene expression levels in this study. Several studies have looked at altered gene expression levels in DS subjects, for example, (Esposito *et al*., 2008; Costa *et al*., 2011; Letourneau *et al*., 2014). (Letourneau *et al*., 2014) showed that the nuclear compartments of trisomic cells undergo modifications of the chromatin environment influencing the overall transcriptome.

To the best of our knowledge, the epigenetic clock approach facilitated the first analysis of DS on age-related epigenetic modifications. Our results indicate that DS can be interpreted as segmental progeria, which affects at least two disparate tissues. As it is well known that DS is associated with clinical manifestations of premature aging in brain and to a lesser extent in blood tissue, it is reassuring that we observe significant age acceleration effects in brain (11 years) and blood (4 years) tissue. Future studies should evaluate whether the extent of epigenetic age acceleration relates to the prevalence of age-related conditions across tissues.

## Supporting Information

**Data S1-S5** Detailed description of datasets, Public availability of data sets, Ethics, DNA methylation measurements, Statistical analysis, Estimation of blood cell type composition based on DNAm data, Reference free method for estimating cell counts and confounders, Evaluation of causal models using structural equation models and Marginal analysis and meta analysis for finding CpGs that relate to DS status or age acceleration. **Fig. s1** Results in different brain regions. **Fig. s2** Results by gender. **Fig. s3** Mean methylation versus age and DS status. **Fig. s4** Blood cell types versus age acceleration in dataset 1. **Fig. s5** Blood cell types versus age acceleration in dataset 3. **Fig. s6** Results for alternative epigenetic biomarkers of aging. **Fig. s7** Preservation of marginal associations with age and age acceleration **Fig. s8** Preservation of associations with DS status. **Fig. s9** Two CpGs that are hyper-methylated in DS. **Fig. s10** Two CpGs that are hypo-methylated in DS. **Table s1** Multivariate regression models involving blood cells. **Table s2** Multivariate regression models based on reference free covariates. **Table s3** Results of the structural equation model analysis.

**Data S6** Results for Supplementary data file for relating CpGs with age acceleration and DS status.
