# Supplementary material for: Accelerated epigenetic aging in Down syndrome
Source: Aging Cell. 2015 Feb 9;14(3):491–5. doi: 10.1111/acel.12325 (PMC4406678; doi:10.1111/acel.12325)
Supplement: Supplementary file 1 [file acel0014-0491-sd1.docx]

**SUPPORTING INFORMATION for the article**

**"Accelerated Epigenetic Aging in Down Syndrome"**

**Horvath et al.**

Contents

[Detailed description of datasets 2](#_Toc404587029)

[Public availability of data sets 4](#_Toc404587030)

[Ethics 4](#_Toc404587031)

[DNA methylation measurements 5](#_Toc404587032)

[Statistical analysis 5](#_Toc404587033)

[Estimation of blood cell type composition based on DNAm data 6](#_Toc404587034)

[Reference free method for estimating cell counts and confounders 6](#_Toc404587035)

[Evaluation of causal models using structural equation models 7](#_Toc404587036)

[Marginal analysis and meta analysis for finding CpGs that relate to DS status or age acceleration 8](#_Toc404587037)

[Supplementary Figure 1. Results in different brain regions. 10](#_Toc404587038)

[Supplementary Figure 2. Results by gender. 11](#_Toc404587039)

[Supplementary Figure 3. Mean methylation versus age and DS status. 12](#_Toc404587040)

[Supplementary Figure 4. Blood cell types versus age acceleration in dataset 1. 13](#_Toc404587041)

[Supplementary Figure 5. Blood cell types versus age acceleration in dataset 3. 14](#_Toc404587042)

[Supplementary Figure 6. Results for alternative epigenetic biomarkers of aging 15](#_Toc404587043)

[Supplementary Figure 7. Preservation of marginal associations with age and age acceleration 16](#_Toc404587044)

[Supplementary Figure 9. Two CpGs that are hyper-methylated in DS 18](#_Toc404587045)

[Supplementary Figure 10. Two CpGs that are hypo-methylated in DS 19](#_Toc404587046)

[Suppl. Table 1. Multivariate regression models involving blood cells 20](#_Toc404587047)

[Suppl. Table 2. Multivariate regression models based on reference free covariates 21](#_Toc404587048)

[Suppl. Table 3. Results of the structural equation model analysis. 22](#_Toc404587049)

[Supplementary data file for relating CpGs with age acceleration and DS status 23](#_Toc404587050)

[REFERENCES 24](#_Toc404587051)

# Detailed description of datasets

**Data set 1**

*Study subjects and diagnosis*: Participants with DS were ascertained through the New York State developmental disability service system as well as agencies in New Jersey, Connecticut and Northern Pennsylvania and have been assessed comprehensively including full medical chart reviews. The participants were recruited through responsible state and private service agencies, who contacted the participant’s families or correspondents for permission for us to recruit. Informed consent was provided by either a parent or correspondent, and assent was obtained from the participant. The distribution of age, level of intellectual disability and residential placement did not differ between those participating and those who refused. Age-matched control participants were laboratory volunteers and participants in the Washington Heights-Inwood Community Aging Project who gave informed consent for genetic studies. Confirmation of trisomy 21 by G-banded karyotypes was available for 98% of the study participants with DS, with 100% concordance between cytogenetics and the clinical diagnosis of DS. Of those karyotyped, the large majority had complete trisomy 21. However, 7 cases exhibited low level mosaicism with most of the cells having trisomy 21 and less than 15% of the cells showing a diploid chromosome complement, 3 cases showed higher level mosaicism with greater than 15 percent of cells having 46 chromosomes (disomic for chromosome 21) and six cases presented with Robertsonian translocations, which in each case produced complete trisomy for the euchromatic region of chromosome 21 in all cells.

**Data set 2**

This novel dataset (measured on the Illumina 450K array) involves brain samples from 15 DS, 25 AD, and 31 normal control samples. The tissues were provided by the Brain tissue and CSF resource/bank of the Mary Easton Alzheimer Disease Research Centre at UCLA (by H. Vinters). The DS samples had a mean age of 49.6 (ranging from 42 to 57). The controls samples had a mean age of 50.5 (ranging from 32 to 64). The AD subjects had a mean age of 60.1 (ranging from 58 to 64). We considered various brain regions: frontal lobe (n=21), cerebellum (n=13), hippocampus (n=9), midbrain (n=6), occipital cortex (n=11), temporal cortex (n=11). In Horvath (2013), we showed that all brain regions have an indistinguishable DNAm age (at least for non-DS subjects). Our results remained qualitatively the same after restricting the analysis to a given brain region.

**Data set 3**

This novel dataset (measured on the Illumina 450K array) involves 29 individuals with Down syndrome, their mothers (DSM) and their unaffected siblings (DSS). This family-based model allowed us to adjust for possible confounding effects on DNA methylation patterns deriving from genetic and environmental (lifestyle) factors. The individuals investigated in this study were recruited in Emilia-Romagna region (Bologna and Ferrara provinces), Italy. Exclusion criteria were current acute illnesses, hepatic, renal or cardiac insufficiency, assumption of antioxidant or nutraceutical substances (vitamins, lipoic acid, acetylcysteine, omega 3 and 6 fatty acids, probiotics) within the last two months. A total of 29 DS (12-43 years, 18 males, 11 females), 29 DSS (9-52 years, 7 males, 22 females) and 29 DSM (42-83 years) were included in the study. All the DS were classified as free trisomy with the exception of 3 translocations and 4 mosaicisms.

DNA extraction and bisulphite treatment of DNA

Extraction of genomic DNA from whole peripheral blood was performed using the QIAamp

96 DNA Blood Kit (QIAGEN, Hilden, Germany). Sodium bisulphite conversion for Infinium HumanMethylation450 BeadChip was performed using the EZDNA Methylation-Gold Kit and the EZ-96 DNA Methylation Kit respectively.

Genome-wide DNA methylation analysis: Genome-wide DNA methylation of 29 families including a DS, a DSM and a DSS was analysed using the Infinium HumanMethylation450 BeadChip (Illumina, San Diego, CA) following manufacturer’s instructions. Arrays were scanned by HiScan (Illumina). GenomeStudio (Illumina) was used to perform background subtraction.

**Data set 4**

These publicly available Illumina 450K data involved buccal epithelial cells from 10 adults with DS and 10 control (Jones et al., 2013). Note that the authors reported that some of the buccal samples from DS subjects were probably contaminated by blood. After removing these contaminated samples (or using a multivariate model for adjusting for confounding) the results became even less significant.

# Public availability of data sets

All data are fully available from NCBI Gene Expression Omnibus (GEO) (<http://www.ncbi.nlm.nih.gov/geo/>). All data sets are publicly available from Gene Expression Omnibus. In particular, novel data sets 2 and 3 have the following GEO accession numbers: GSE63347, GSE52588.

# Ethics

The subjects from dataset 3 were recruited from the Emilia-Romagna region (Bologna and Ferrara provinces), Italy, with the help of non-profit associations CEPS, OPIMM and ANFFAS. The study was approved by the local Ethical Committee (S. Orsola Hospital, University of Bologna; Prot. n. 126/2007/U/Tess, 18/12/2007). A written informed consent form was obtained from parents and relatives of DS and from adult DS.

Brain data set 2: All subjects from the UCLA tissue bank signed the "Consent for Autopsy" form by the Department of Pathology at UCLA. Further, the epigenetic analysis is covered by IRB Research Protocol Number: 19119.

# DNA methylation measurements

In brief, bisulﬁte conversion using the Zymo EZ DNA Methylation Kit (ZymoResearch, Orange, CA, USA) as well as subsequent hybridization of the HumanMethylation450K Bead Chip (Illumina, SanDiego, CA), and scanning (iScan, Illumina) were performed according to the manufacturers protocols by applying standard settings. DNA methylation levels (β values) were determined by calculating the ratio of intensities between methylated (signal A) and un-methylated (signal B) alleles. Specifically, the β value was calculated from the intensity of the methylated (M corresponding to signal A) and un-methylated (U corresponding to signal B) alleles, as the ratio of fluorescent signals β = Max(M,0)/[Max(M,0) +Max(U,0) + 100]. Thus, β values range from 0 (completely un-methylated) to 1 (completely methylated) (Dunning et al., 2008).

# Statistical analysis

Epigenetic age (also known as DNAm age) was calculated as reported previously (Horvath, 2013a). The epigenetic clock is defined as a prediction method of age based on the DNAm levels of 353 CpGs. The 353 CpGs of the epigenetic clock were identified using a penalized regression model approach (elastic net) that regressed chronological age on roughly 21k CpGs in independent data (which were not used in the current article). Predicted age is referred to as DNAm age. Mathematical details and software tutorials for the epigenetic clock can be found in the additional files (supplements) of (Horvath, 2013a). An online age calculator can be found at our webpage (Horvath, 2013b). Many authors have described methods for dealing with the two types of probes found on the Illumina 450K array (Maksimovic et al., 2012; Teschendorff et al., 2013; Yousefi et al., 2013). This is not a concern for the epigenetic clock which applies to both Illumina platforms. But the epigenetic clock software implements a data normalization step that repurposes the BMIQ normalization method from Teschendorff (Teschendorff et al., 2013) so that it automatically references each sample to a gold standard based on mean methylation levels in a training data set. Additional details can be found in Additional file 2 from (Horvath, 2013a).

# Estimation of blood cell type composition based on DNAm data

We used the Houseman algorithm for inferring blood cell counts from the Illumina 27K methylation data (Houseman et al., 2012). Further, we used an adaptation of this method to Illumina 450K data implemented in the minfi R package (Jaffe and Irizarry, 2014).

# Reference free method for estimating cell counts and confounders

The Houseman algorithm for estimating the cell type composition requires reference datasets which may be hard to come by (e.g. for our brain and buccal cells). Even for blood cells, potentially subtle shifts in blood cell populations may not be captured by the reference libraries of major cell types. For example, there could be natural killer cell activation states that are really driving the observed changes in DNAm levels. To address these issues, Houseman (2014) recently proposed the "RefFreeEWAS" method for conducting epigenome-wide association studies analysis when a reference dataset is unavailable (Houseman et al., 2014). Following a personal communication from A. Houseman, we adapted this method as follows. We applied the singular value decomposition (SVD) to the untransformed methylation data and then used the first *K* left-singular vector in the multiple regression model (Suppl. Table 2) prediction model. Following the recommendation from Houseman (2014) and Teschendorff (2011) (Teschendorff et al., 2011) we selected the dimension *K* using random matrix theory (RMT) using the R function "*EstDimRMT*". Although *K* was estimated to be one in all models, we chose a larger number (*K*=3) in order to err on the side of caution. The resulting 3 singular vectors (denoted SV1, SV2, SV3 in Suppl. Table 2) were used as additional covariates in the multiple regression model. Although our moderate sample size did not allow us to fit many covariates, it is worth mentioning that our results are largely unchanged after including both estimated blood cell counts and singular vectors in the same multiple regression model.

# Evaluation of causal models using structural equation models

In the following, we discuss several causal models that could explain the relationship between DS status, age acceleration, and the proportion of a given blood cell type.

Model 1 (DS -> abundance of blood cell type -> age acceleration)

Model 1 posits that the DS acts on age acceleration through changes in blood cell counts. In other words, accelerated age effects due to DS are mediated by changing numbers of blood cell counts. Although this model is biologically plausible, it does not fit our blood datasets (Supplementary Table 2).

Model 2 (DS -> age acceleration -> abundance of blood cell type)

Model 2 posits that DS causes an increase in age acceleration which subsequently explains the observed changes in blood cell composition. This model fits some of the data sets (Supplementary Table 2).

Model 3: (abundance of blood cell type <- DS -> age acceleration)

Model 3 is the independence model, in which DS causes changes in blood cell type abundance and age acceleration independently. In other words, in this model DS confounds the relationship between blood cell counts and age acceleration. This model fits most of the data sets (Supplementary Table 2).

Model 4: (DS -> abundance of blood cell type <- age acceleration)

Model 4 posits that both DS and age acceleration lead to changes in blood cell composition. This model does not fit the data (Supplementary Table 2).

Model 5: (DS -> age acceleration <- abundance of blood cell type)

Model 5 posits that both DS and changes in blood cell composition explain the observed age acceleration effect. This model fits some of the data sets (Supplementary Table 2).

**Interpretation of the structural equation model analysis**

We caution the reader that structural equation models make several assumptions (reviewed in (Aten et al., 2008; Fox, 1984, 2006; Kline, 2005; Pearl, 1988; Shipley, 2000)) which may not be satisfied in our data. We used the *sem* R package (Fox, 2006) to evaluate the fit of causal models. The sem R function provides the model fitting chi-square statistic which was used to compute a model fitting p-value for each causal model. For example, P(data|DS → CD4 → AA) denotes the p-value for the model in which DS causally affects the proportion of CD4 T cells which in turn affects age acceleration. The model fitting chi-square statistic tests the null hypothesis that the model is correct. A small model p-value (say p < 0.05) indicates that the causal model does not fit well. Following the logic of an 'accept-support' context (Kline, 2005; Steiger and Fouladi, 1997) where the null hypothesis represents the researchers belief, it is the failure to reject the null hypothesis that supports the causal model. The model fit statistic and the corresponding model p-value have several limitations, e.g. they are sensitive to the size of correlations and they depend on the sample size N (Kline, 2005). However, the model fitting p-value is the key ingredient of most, if not all, alternative model fitting indices.

# Marginal analysis and meta analysis for finding CpGs that relate to DS status or age acceleration

Our meta analysis pursued two goals: 1) To identify CpGs that are associated with DS status in data sets brain and blood data (data sets 1-3). 2) To identify CpGs that are associated with epigenetic age acceleration in brain and blood (data sets 1-3). In order to combine data sets 1-3, we focused on the roughly 26k CpGs that are shared between the Illumina 27K and the 450K platforms. A detailed marginal analysis of all CpGs will be presented elsewhere. Or the reader can carry out this analysis after downloading our publicly available data. To relate CpGs to DS status in data sets 1, 2, 4, we used the Kruskal Wallis test since it is a non-parametric group comparison test. In data set 3, we used a linear mixed effects model since this data set is comprised of families (including discordant sib pairs).

To identify CpGs that correlate with age acceleration, we used a robust correlation test: the biweight midcorrelation coefficient implemented in the R function "*standardScreeningNumericTrait*" from the WGCNA R library (Langfelder and Horvath, 2008).

The resulting p-values were log transformed (base 10) and multiplied by a sign so that a positive value corresponds to a positive correlation coefficient. Note that these log transformed p-values can be used to rank the CpGs in each data sets. The R function "*rankPvalue*" (Langfelder et al., 2013) implemented in the WGCNA R package was used to calculates p-values for observing that a CpG has a consistently high or low ranking according to multiple log p-values (corresponding to the different data sets). Specifically, the rankPvalue function results in two meta analysis p-values "*pValueHighRank*" and "*pValueLowRank*" which assess the probability of observing a consistently high or low ranking. The 2-sided meta analysis p-value was defined as two times the minimum of pValueHighRank and pValueLowRank. The log (base 10) transformed meta analysis p-value was multiplied by -1 for CpGs with pValueLowRank< pValueHighRank.

To find CpGs that relate to age acceleration we used the first 3 data sets (all brain and blood data sets). To identify CpGs that relate to DS status, we used the first 3 data sets and the frontal cortex samples from data set 2 to ensure that the resulting CpGs are not confounded by brain region.

# Supplementary Figure 1. Results in different brain regions.

Columns correspond to different subsets of our brain DNA methylation data (data set 2). (a,e) is identical to the second column of Figure 1, i.e. it reports the results for all brain samples. The second column (b,f) and third column (c,g) report the corresponding results when the analysis is restricted to samples from the cerebellum and frontal lobe, respectively. The fourth column (d,h) reports findings for the other brain regions (i.e. samples from the hippocampus, midbrain, occipital cortex, and temporal cortex). The orange line corresponds to a regression line through DS subjects. The black line in the upper panels indicates the regression line through the remaining (control) samples. The age acceleration effect for each subject (point) corresponds to the vertical distance to the black regression line. (a-d) DNAm age (y-axis) versus chronological age in the respective data sets. Points are colored by disease status: red for DS, blue for AD, and black for controls. The bar plots in the second row (e-h) show how mean age acceleration relates disease status. The bar plots also report a Kruskal Wallis p-value and one standard error around the mean value.

# Supplementary Figure 2. Results by gender.

The panels are similar to those in Figure 1 but the analysis is stratified by gender. The scatter plots in the first row (a-c) show how DNAm age (y-axis) relates to chronological age in females samples only. Analogous plots for males can be found in the third row (g-i). Points are colored by disease status: red for DS, blue for AD, and black for controls. Age acceleration is defined as residual from the regression line through all points. The bar plots in the second (d-f) and fourth row (j-l) show that mean age acceleration (y-axis) is significantly related to DS status (x-axis) when restricting the analysis to females from dataset 1 (d) or males from dataset 2 (k) or data set 3 (l). The insignificant results for males from datasets 1 (j) probably reflects the low sample sizes (only 5 DS cases). The bar plots also report the Kruskal Wallis test p-value and 1 standard error around the mean value. Data set 4 was excluded in this stratified analysis because of its low sample size.

# Supplementary Figure 3. Mean methylation versus age and DS status.

The scatter plots in the first row and third rows (a-c, g-i) show the relation between mean methylation (across the 26k CpGs that are present on both the Illumina 27K and the 450K platform) and chronological age in each dataset/gender combination. The dashed red line corresponds to the regression line based on both DS subjects and controls. The bar plots in the second row (d-f) and fourth row (j-l) show that mean methylation (y-axis) is not significantly related to DS status or AD status (x-axis) at a 0.05 level. Points are colored by disease status: red for DS, blue for AD, and black for controls. The caption of each bar plot reports the Kruskal Wallis test p-value and 1 standard error around the mean. Data set 4 was excluded because of its low sample size.

# Supplementary Figure 4. Blood cell types versus age acceleration in dataset 1.

(a-f) Age acceleration versus proportion of CD8 T cells (a), CD4 T cells (b), Natural Killer (c), B cells (d), monocytes (e), and granulocytes (f) in all samples from dataset 1. DS cases and controls correspond to red circles and black squares respectively. Age acceleration does not exhibit a significant correlation with cell proportions according to a robust correlation test (based on the biweight midcorrelation correlation). (g-l) Analogous plots in control subjects only. Only the proportion of monocytes exhibits a significant correlation with age acceleration (k). (m-r) Proportion of cell types versus DS status. DS status is not significantly associated with cell type proportions according to the Kruskal Wallis test.

# Supplementary Figure 5. Blood cell types versus age acceleration in dataset 3.

(a-f) Age acceleration versus proportion of CD8 T cells (a), CD4 T cells (b), Natural Killer (c), B cells (d), monocytes (e), and granulocytes (f) in dataset 3. DS cases and controls correspond to red circles and black squares respectively. Age acceleration has a significant negative correlation with the proportion of CD4 T cells and monocytes across all samples. (g-l) analogous plots in control subjects. None of the correlations are significant in the control samples. (m-r) Proportion of cell types versus DS status. DS status is significantly associated with the proportion of CD4 T cells (n), NK cells (o), and B cells (p).

# Supplementary Figure 6. Results for alternative epigenetic biomarkers of aging

Here we show that accelerated aging effects due to DS can also be observed using CpGs that are part of three aging signatures reported in the literature. The columns correspond to the 4 data sets. Panels in the first row (a-d) describe the performance of the age predictor described in Hannum et al (2013)(Hannum et al., 2013). Note that it can only be applied to dataset 3 (c) since it does not apply to data generated on the Illumina 27K platform (a), to brain tissue (b), or buccal epithelium (d). (e-l) The bar plots report the mean Student T-test statistic for testing the relationship between methylation levels and DS status for groups of CpGs that make up the aging signatures. Each bar corresponds to a group of CpGs. (e-h) involve the group of 1000 age related CpGs from our previous publication (Horvath et al 2012) (Horvath et al., 2012). (i-l) involve a group of 213 CpGs from Teschendorff et al 2010 (Teschendorff et al., 2010) that become hyper methylated with age. The highly significant results in first three data sets (e,f,g,i,j,k) show that CpGs that are part of either aging signature have significantly higher (positive) T statistics than the remaining CpGs, i.e. DS subjects look older than control subjects in terms of these aging signatures.


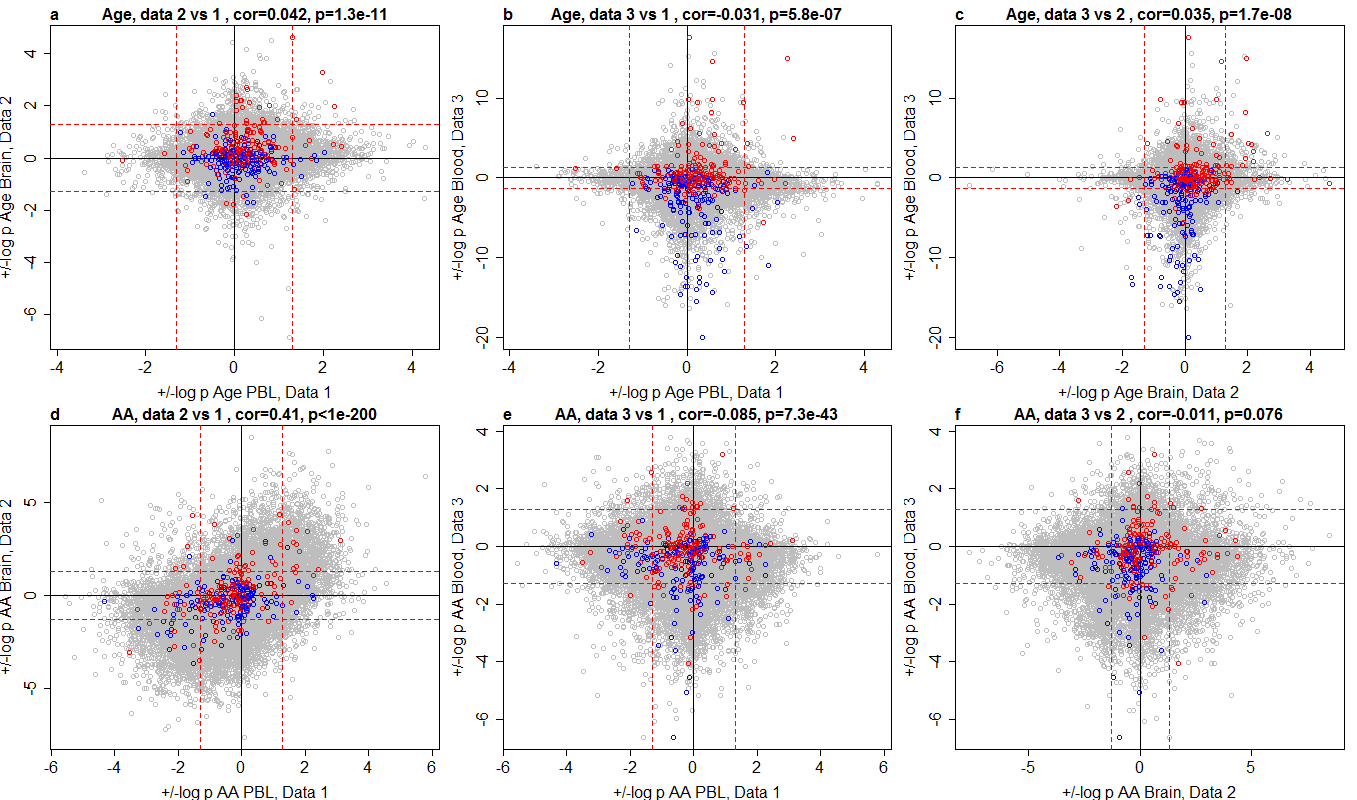


# Supplementary Figure 7. Preservation of marginal associations with age and age acceleration

a-c) CpGs were correlated with chronological age in the blood and brain data sets. Each axis reports the signed logarithm (base 10) transformed correlation test p-value in the respective data set. The sign of the log-transformed p-value was chosen so that a positive (negative) value indicates that the CpG has a positive (negative) correlation with age. The 353 CpGs that make up the epigenetic clock are colored by age relationship in the original training data set from (Horvath, 2013): red and blue correspond to clock CpGs with a positive and negative age correlation, respectively. Signed log p-value in a) brain data set 2 versus PBL data set 1, b) blood data set 3 vs. data set 1, c) data set 3 vs. data set 2. The dashed red horizontal and vertical lines correspond to an uncorrected p-value threshold of 0.05. The weak positive and negative pairwise correlations in the figure titles indicate that age effects are not preserved in these data. Overall, these results suggest that the total (epigenetic clock) is more than the sum of its parts. d-f) analogous plots for age acceleration. We focused on the roughly 26k CpGs that are shared between the two Illumina platforms.


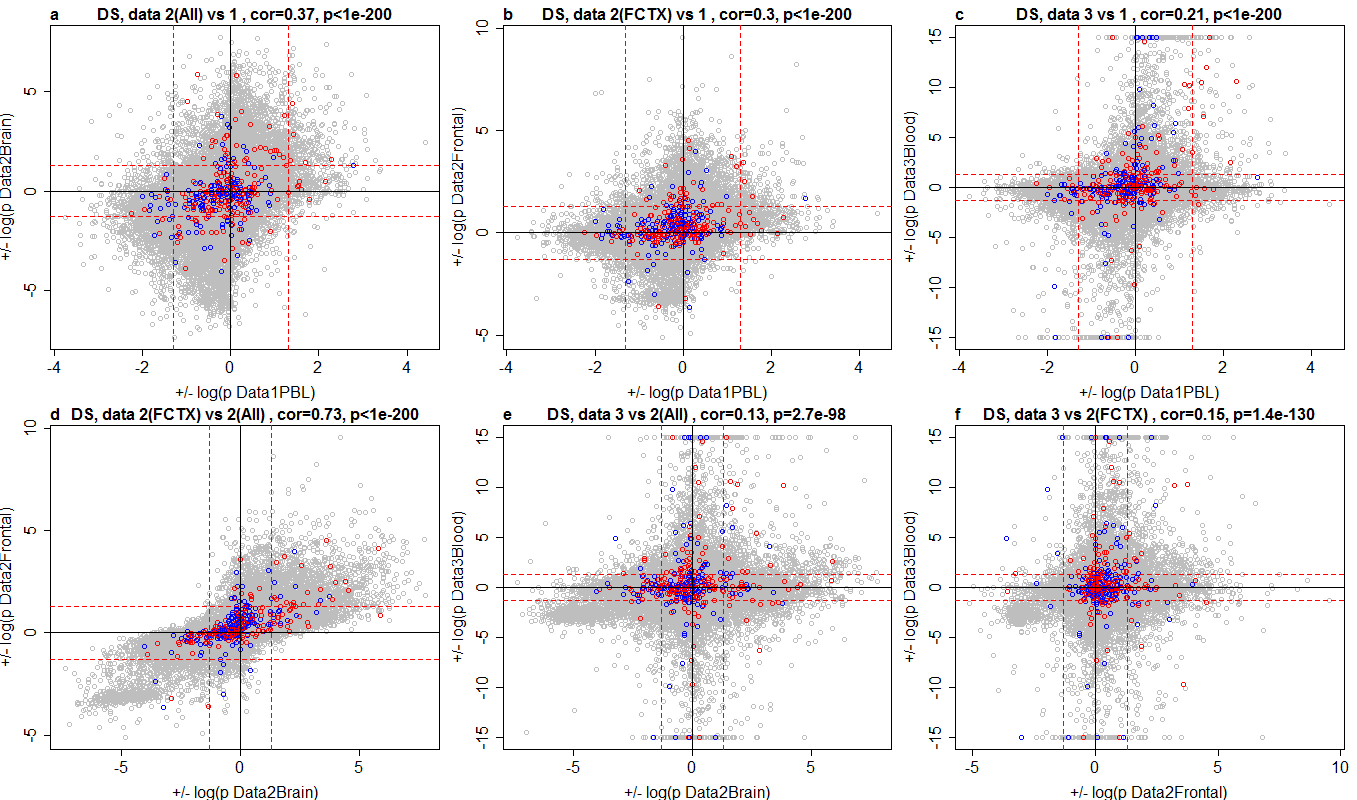
Supplementary Figure 8. Preservation of associations with DS status CpGs were related to Down syndrome status using a 2 group comparison test. Each axis reports the signed logarithm (base 10) transformed p-value in the respective data set. The sign of the log-transformed p-value was defined such that a positive value indicates that the CpG is hyper-methylated in DS subjects. Signed log p-value in a) brain data set 2 versus PBL data set 1, b) frontal cortex samples from data set 2 vs. data set 1, c) blood data set 3 vs. data set 1, d) frontal cortex vs. all brain samples from data set 2, e) blood data set 3 vs. data set 2, f) data set 3 vs. frontal cortex samples from data set 2. The moderate pairwise correlations in the figure titles indicate that DS associations of individual clock CpGs are moderately preserved across the data sets. The 353 clock CpGs are colored according to their age correlation in the original training data set from (Horvath, 2013a): red and blue for clock CpGs that have a positive and negative age correlation, respectively. The red horizontal and vertical lines correspond to an uncorrected p-value threshold of 0.05. Note that relatively few clock CpGs have a significant association with DS status. Statistical details: the Kruskal Wallis test was used in data sets 1 and 2. A linear mixed effects model was used to regress DNAm levels on DS status in family data set 3. Since some of the resulting log p values were almost infinite we imposed an upper bound of +/-15. We focused on the roughly 26k CpGs that are shared between the two Illumina platforms.

Supplementary Figure 9. Two CpGs that are hyper-methylated in DS Results for two individual CpGs that are both hyper-methylated in DS subjects and have a positive correlation with age acceleration (corresponding to the two magenta triangles in the upper right hand corner of Figure 2d). The first two rows (a-f) present findings for CpG cg12186917 which is located in the shore of a CpG island near gene CRYZL1 (crystallin; zeta-like 1) on chromosome 21. The last two rows (g-l) present findings for CpG cg04455759 which is located in the shore of a CpG island near gene MRPL42 (mitochondrial ribosomal protein L42 isoform a) on chromosome 12. Both CpGs are significantly hyper methylated in DS subjects, as can be seen from the bar plots in the first (a-c) and third row (g-i), respectively. The bar plots show the mean methylation value (and one standard error) and report the results from a non-parametric group comparison test (Kruskal Wallis test). Both CpGs have a significant positive correlation with age acceleration as can be seen from the second (d-f) and fourth row (j-l), respectively. Incidentally, these CpG are neither significantly correlated with chronological age nor part of the epigenetic clock.

Supplementary Figure 10. Two CpGs that are hypo-methylated in DS

Results for two individual CpGs that are both hypo-methylated in DS subjects and have a negative correlation with age acceleration. These CpGs correspond to the green triangles in the lower left corner of Figure 2d. The first two rows (a-f) present findings for CpG cg11678767 which is located outside of a CpG island near COL4A6 (type IV alpha 6 collagen isoform A precursor) on the X chromosome. The last two rows (g-l) present findings for CpG cg20425293 which is located in the shore of a CpG island near gene SDHD (succinate dehydrogenase complex; subunit D precursor) on chromosome 11. The bar plots (a-c, g-i) show the mean methylation value (+/- one standard error) and report the results from a non-parametric group comparison test (Kruskal Wallis test). Both CpGs have a significant negative correlation with age acceleration as can be seen from the second (d-f) and fourth row (j-l), respectively. Incidentally, these CpG are neither significantly correlated with chronological age nor part of the epigenetic clock.

# Suppl. Table 1. Multivariate regression models involving blood cells

In multivariate regression models of DNAm age, chronological age and DS status remain significant even after including measures of blood cell type abundance. DS status is a significant predictor of DNAm age in all datasets. The table reports estimates of the regression coefficients and corresponding standard errors, Wald test p-values. The last row reports the age acceleration associated with DS status. For example, DS is associated with an increase of 4.0 years (=3.68976 /0.93453) in DNAm age in dataset 1. A linear mixed effects model was used to the discordant sib pair data of dataset 3 in order to account for relatedness.

|  | Data set 1 | | Data set 3 | | |
| --- | --- | --- | --- | --- | --- |
| **Variable** | **Estimate**  **(Std Err)** | **p** | **Estimate**  **(Std Err)** | | **p** |
| Chron. age | 0.93453  (0.06574) | < 2×10^-16^ | 0.84638 (0.0537) | < 2×10^-16^ | |
| DS status | 3.68976 (1.4702) | 0.016 | 3.413032 (1.30192) | 8.7×10^-3^ | |
| CD8 T | 54.75334 (51.7937) | 0.30 | 7.379152 (39.894) | 0.85 | |
| CD4 T | -60.74226 (51.7714) | 0.25 | -13.90463 (34.1095) | 0.68 | |
| NK | -48.75937 (51.4452) | 0.35 | -8.655011 (35.78581) | 0.81 | |
| B cell | -55.14453 (52.2609) | 0.30 | -18.01050 (44.99109) | 0.69 | |
| Monocytes | -24.50074 (51.4744) | 0.25 | -62.49421 (40.23251) | 0.12 | |
| Granulocytes | -59.9217 (51.474) | 0.25 | -5.567316 (34.78268) | 0.87 | |
| R-squared | 0.85 |  |  |  | |
| Age accel. for DS | 4.0 yrs |  | 4.0 yrs |  | |

# Suppl. Table 2. Multivariate regression models based on reference free covariates

In multivariate regression models of DNAm age, chronological age and DS status remain significant even after including measures of the reference free analysis. DS status is a significant predictor of DNAm age in all datasets. The table reports estimates of the regression coefficients and corresponding standard errors, Wald test p-values. The last row reports the age acceleration associated with DS status. A linear mixed effects model was used to the discordant sib pair data of dataset 3 in order to account for relatedness.

|  | **Data set 1 (PBL)** | | **Data set 2 (Brain)** | | **Data set 3 (Blood)** | | | **Data set 4 (Buccal)** | |
| --- | --- | --- | --- | --- | --- | --- | --- | --- | --- |
| **Variable** | Estimate  (Std Err) | p | Estimate  (SE) | p | Estimate  (SE) | p | | Estim.  (SE) | p |
| Chron. age | 0.927  (0.0627) | <2×10^-16^ | 0.735 (0.058) | <2×10^-16^ | 0.841 (0.053) | <2×10^-16^ | | 0.775 (0.135) | 5.1×10^-5^ |
| DS status | 3.53 (1.39) | 0.014 | 7.269 (1.68) | 5.3x10^-5^ | 2.30 (1.20) | 0.05 | | -0.524 (1.87) | 0.78 |
| SV1 | -316 (315) | 0.32 | -237 (176) | 0.18 | 440  (273) | 0.11 | | -89.5 (213) | 0.68 |
| SV2 | 3.880 (4.93) | 0.43 | 3.765 (4.52) | 0.41 | 10.1 (5.04) | 0.046 | | 11.1 (3.88) | 0.012 |
| SV3 | 4.594 (6.44) | 0.48 | 3.560 (5.27) | 0.50 | -6.44 (5.76) | 0.26 | | -6.59 (3.53) | 0.083 |
| R^2 | 0.85 |  | 0.74 |  |  |  | | 0.85 |  |
| Age accel DS | 3.8 yrs |  | 9.9 | | 2.7 |  |  | -0.7 |  |

# Suppl. Table 3. Results of the structural equation model analysis.

The table reports model fitting p-values based on an SEM analysis. Rows correspond to the two blood datasets and columns to different causal scenarios between DS status, proportion of the blood cell type, and age acceleration (AA). The p-values correspond to a likelihood ratio test of the null hypothesis that the data fit the causal model. Model fitting p-values that are significant at a 0.05 threshold (colored in red and marked by ^†^) correspond to causal models that do not fit the data. In contrast, model fitting p-value larger than 0.15 (colored in green and marked by an asterisk *) corresponds to models that fit the data. None of the causal models that posit that changes in blood cell type abundance mediate the effect of DS on age acceleration (model 1) fit the data.

|  | **Causal models** | | | | |
| --- | --- | --- | --- | --- | --- |
|  | **Model 1** | **Model 2** | **Model 3** | **Model 4** | **Model 5** |
| **Data Set** | **DS->CD4->AA** | **DS->AA->CD4** | **CD4<-DS->AA** | **DS->CD4<-AA** | **DS->AA<-CD4** |
| 1 | 6.5E-3^†^ | 9.1E-1* | 1.8E-1* | 6.0E-3^†^ | 7.1E-1* |
| 3 | 1.1E-2 | 5.1E-5^†^ | 7.2E-1* | 1.4E-3^†^ | 7.0E-6^†^ |
|  | **DS->CD8->AA** | **DS->AA->CD8** | **CD8<-DS->AA** | **DS->CD8<-AA** | **DS->AA<-CD8** |
| 1 | 5.9E-3^†^ | 8.6E-1* | 5.4E-1* | 6.0E-3^†^ | 9.7E-1* |
| 3 | 2.6E-3^†^ | 5.5E-1* | 1.9E-1* | 1.4E-3^†^ | 2.2E-1* |
|  | **DS->Bcell->AA** | **DS->AA->Bcell** | **Bcell<-DS->AA** | **DS->Bcell<-AA** | **DS->AA<-Bcell** |
| 1 | 6.7E-3^†^ | 7.0E-2 | 8.6E-1* | 6.0E-3^†^ | 6.2E-2 |
| 3 | 6.4E-3^†^ | 3.2E-2 | 3.2E-1* | 1.4E-3^†^ | 6.5E-3^†^ |
|  | **DS->Mono->AA** | **DS->AA->Mono** | **Mono<-DS->AA** | **DS->Mono<-AA** | **DS->AA<-Mono** |
| 1 | 6.8E-3^†^ | 8.3E-1* | 4.0E-2^†^ | 6.0E-3^†^ | 6.0E-1* |
| 3 | 9.9E-5^†^ | 1.4E-2^†^ | 6.0E-4^†^ | 1.4E-3^†^ | 3.0E-1* |

# Supplementary data file for relating CpGs with age acceleration and DS status

The zipped comma delimited file "SupplementaryFileMarginalCpG.zip" reports the results for a marginal analysis of the roughly 26k CpGs that are shared between the Illumina 27K and 450K platform. Details can be found in the section entitled "*Marginal analysis and meta analysis for finding CpGs that relate to DS status or age acceleration*". Briefly, each CpG was related to a) epigenetic age acceleration (defined as residual from a regression line) and b) DS status. CpG denotes the probe name on the Illumina array. GeneSymbol= gene symbol for a neighboring gene. ClockCpG= indicates whether a CpG is one of the 353 CpGs that make up the epigenetic clock. corAAData1PBL=correlation coefficient between epigenetic age acceleration (AA) and DNA methylation levels. The correlation coefficient was calculated using the biweight midcorrelation coefficient implemented in the R function *standardScreeningNumericTrait* from the WGCNA R library (Langfelder and Horvath, 2008). signedLogAAData1PBL= signed log-transformed (base 10) p-value of the correlation test p-value. p.metaAgeAccelFirst3dataHighRank=meta analysis p-value for age acceleration based on the brain and blood sets (i.e. the first 3 data sets). Specifically, it is the 1-sided p-value "pValueHighRank" resulting from the "rankPvalue function" in the WGCNA R library. p.metaAgeAccelFirst3dataLowRank=analogous meta analysis p-value based on pvalueLowRank. signedLog10.p.metaAgeAccel= signed log-transformed (base 10) meta analysis p-value for age acceleration across the first 3 data sets. PData1PBL.DS= Two sided Kruskal Wallis test p-value for relating DS status with the CpG in data set 1. p.LME.Data3Blood.DS= Asymptotic p-value resulting from a linear mixed effects model that regressed DNAm levels on disease status but adjusted for family relationships in data set 3. p.metaDSFirst3dataAndFCTXHighRank=meta analysis p-value for DS status based on the first 3 data sets and the frontal cortex samples from data set 2. All of the meta p-values should be interpreted as a descriptive (as opposed to inferential) measures.

# REFERENCES

Aten, J., Fuller, T., Lusis, A., and Horvath, S. (2008). Using genetic markers to orient the edges in quantitative trait networks: The NEO software. BMC systems biology *2*, 34.

Dunning, M., Barbosa-Morais, N., Lynch, A., Tavare, S., and Ritchie, M. (2008). Statistical issues in the analysis of Illumina data. BMC Bioinformatics *9*, 85.

Fox, J. (1984). "Linear Structural-Equation Models", Vol chapter 4 (NY: Wiley).

Fox, J. (2006). Structural Equation Modeling With the sem Package in R. Structural Equation Modeling *13*, 465 - 486.

Hannum, G., Guinney, J., Zhao, L., Zhang, L., Hughes, G., Sadda, S., Klotzle, B., Bibikova, M., Fan, J.-B., Gao, Y.*, et al.* (2013). Genome-wide Methylation Profiles Reveal Quantitative Views of Human Aging Rates. Molecular cell *49*, 359-367.

Horvath, S. (2013a). DNA methylation age of human tissues and cell types. Genome Biol *14*.

Horvath, S. (2013b). Webpage: <http://labs.genetics.ucla.edu/horvath/dnamage>.

Horvath, S., Zhang, Y., Langfelder, P., Kahn, R., Boks, M., van Eijk, K., van den Berg, L., and Ophoff, R.A. (2012). Aging effects on DNA methylation modules in human brain and blood tissue. Genome Biology *13*, R97.

Houseman, E., Accomando, W., Koestler, D., Christensen, B., Marsit, C., Nelson, H., Wiencke, J., and Kelsey, K. (2012). DNA methylation arrays as surrogate measures of cell mixture distribution. BMC Bioinformatics *13*, 86.

Houseman, E.A., Molitor, J., and Marsit, C.J. (2014). Reference-free cell mixture adjustments in analysis of DNA methylation data. Bioinformatics *30*, 1431-1439.

Jaffe, A., and Irizarry, R. (2014). Accounting for cellular heterogeneity is critical in epigenome-wide association studies. Genome Biology *15*, R31.

Jones, M.J., Farre, P., McEwen, L.M., Macisaac, J.L., Watt, K., Neumann, S.M., Emberly, E., Cynader, M.S., Virji-Babul, N., and Kobor, M.S. (2013). Distinct DNA methylation patterns of cognitive impairment and trisomy 21 in Down syndrome. BMC Med Genomics *6*, 58.

Kline, R. (2005). Principles and Practice of Structural Equation Modeling. (New York, NY: The Guilford Press).

Langfelder, P., and Horvath, S. (2008). WGCNA: an R package for weighted correlation network analysis. BMC Bioinformatics *9*, 559.

Langfelder, P., Mischel, P.S., and Horvath, S. (2013). When is hub gene selection better than standard meta-analysis? PLoS ONE *8*, e61505.

Maksimovic, J., Gordon, L., and Oshlack, A. (2012). SWAN: Subset-quantile Within Array Normalization for Illumina Infinium HumanMethylation450 BeadChips. Genome Biology *13*, R44.

Pearl, J. (1988). Probabilistic reasoning in intelligent systems: networks of plausible inference., 2 edn (San Francisco, CA: Morgan Kaufmann Publishers, Inc).

Shipley, B. (2000). Cause and Correlation in Biology. 2nd edition. , 2 edn (Cambridge, UK: Cambridge University Press).

Steiger, J., and Fouladi, R. (1997). Noncentrality interval estimation and the evaluation of statistical models. (Erlbaum, Mahwah, NJ).

Teschendorff, A.E., Marabita, F., Lechner, M., Bartlett, T., Tegner, J., Gomez-Cabrero, D., and Beck, S. (2013). A beta-mixture quantile normalization method for correcting probe design bias in Illumina Infinium 450 k DNA methylation data. Bioinformatics *29*, 189-196.

Teschendorff, A.E., Menon, U., Gentry-Maharaj, A., Ramus, S.J., Weisenberger, D.J., Shen, H., Campan, M., Noushmehr, H., Bell, C.G., Maxwell, A.P.*, et al.* (2010). Age-dependent DNA methylation of genes that are suppressed in stem cells is a hallmark of cancer. Genome research *20*, 440-446.

Teschendorff, A.E., Zhuang, J., and Widschwendter, M. (2011). Independent surrogate variable analysis to deconvolve confounding factors in large-scale microarray profiling studies. Bioinformatics *27*, 1496-1505.

Yousefi, P., Huen, K., Schall, R.A., Decker, A., Elboudwarej, E., Quach, H., Barcellos, L., and Holland, N. (2013). Considerations for normalization of DNA methylation data by Illumina 450K BeadChip assay in population studies. Epigenetics *8*, 1141-1152.
